# Grandparental socioeconomic disadvantages and grandchild psychiatric disorders: the mediating role of parental socioeconomic and psychosocial factors

**DOI:** 10.1038/s41598-025-04282-z

**Published:** 2025-06-20

**Authors:** Baojing Li, Can Liu, Ylva B. Almquist, Lisa Berg

**Affiliations:** Department of Public Health Sciences, Centre for Health Equity Studies (CHESS), Stockholm University, SE-106 91, Stockholm, Sweden

**Keywords:** Socioeconomic factors, Psychosocial factors, Mental health, Multigenerational transmission, Mediation, Sweden, Risk factors, Psychology and behaviour, Socioeconomic scenarios

## Abstract

The aim of this study is to explore the association between grandparental socioeconomic disadvantages and grandchild psychiatric disorders, the role of parental socioeconomic and psychosocial factors in this association, as well as potential gender differences. We utilized a cohort study design using data from the Stockholm Birth Cohort Multigenerational Study, including 11,299 individuals born in 1953 (parental generation), their 22,598 parents (grandparental generation), and 24,707 adult children (grandchild generation). Grandparental and parental socioeconomic disadvantages, respectively, included low income, non-employment, and overcrowding. Parental psychosocial disadvantages included single parenthood, psychiatric disorders, and criminality. Psychiatric disorders in the grandchildren were reflected by hospitalizations due to mental and behavioral disorders from age 18 to 30 (1986–2019). Analyses were performed within the Structural Equation Modeling framework. We found an association between grandparental socioeconomic disadvantages and grandchild psychiatric disorders (standardized total effect 0.155, 95% confidence interval [CI] 0.099–0.211), which was mediated through parental psychosocial disadvantages (standardized mediating effect 0.101, 95% CI 0.073–0.130). The mediation was more pronounced via psychosocial disadvantages among mothers than fathers. These findings indicate that psychosocial disadvantages among parents, especially mothers, reflect an important mediating mechanism, and addressing such disadvantages may help mitigate social inequalities in mental health across generations.

## Introduction

Psychiatric disorders are often recognized to have a genetic basis, but environmental influences such as social and economic resources also influence the occurrence of these disorders^1^. More recently, based on abundant evidence on associations between family socioeconomic conditions and psychiatric disorders in offspring^2^, increasing research interest has shifted focus to the effects of socioeconomic conditions on third-generation psychiatric outcomes, including, for example, grandchild eating disorders^3^, depression^4^, alcohol-related disorders^5^, and behavior problems^6^. In general, a scoping review of multigenerational impacts of grandparental exposures on grandchild mental health has concluded that psychiatric disorders may be outcomes of unfavorable exposures originating from the grandparental generation^7^.

However, studies on the multigenerational association between grandparental socioeconomic conditions such as school performance, education, and family resources^3,4,6^ and grandchild psychiatric outcomes have so far overlooked the exploration of mechanisms—in other words, the indirect effects through multiple factors in the intermediate generation of parents. Evidence from previous intergenerational studies supports the possible existence of such indirect effects by demonstrating associations between these factors among parents and offspring psychiatric outcomes, as well as between parental socioeconomic disadvantages and these factors among offspring. These factors, broadly categorized into domains of socioeconomic (e.g., poverty, low income, unemployment, residential crowding, and receipt of social assistance) and psychosocial disadvantages (e.g., single parenthood, mental health problems, crime and imprisonment), can be both consequences of socioeconomic disadvantages from the family of origin^2,8–11^, and predictors for psychiatric outcomes among the offspring^12–16^.

To our knowledge, no longitudinal study has addressed the hypothesis that grandparental socioeconomic disadvantages might give rise to grandchild psychiatric disorders, potentially operating through multiple factors in the parental generation. This hypothesis is underpinned by cumulative inequality theory, which emphasizes that (dis)advantages are transmitted and consolidated across multiple generations^17^. In addition, less is known about whether socioeconomic or psychosocial aspect of these factors play a more important role in the underlying mechanisms. Furthermore, potential gender differences are also relevant for investigation^18^. For example, previous studies suggest that family social and economic resources predict depressive symptoms only among young adult men^19^, and intergenerational transmission of emotional disorders seems to be stronger among mothers and daughters^20^.

Against this background, we aim to explore the association between socioeconomic disadvantages of grandparents and psychiatric disorders of their grandchildren, as well as the role of socioeconomic and psychosocial factors in the parental generation. Additionally, we examine potential differences by gender across the parental and grandchild generations. We address the following research questions:

1. Is there an association between socioeconomic disadvantages (i.e., low income, non-employment, and household overcrowding) among grandparents and psychiatric disorders among grandchildren?
2. To what extent is the association between grandparental socioeconomic disadvantages and grandchild psychiatric disorders mediated through socioeconomic (i.e., low income, non-employment, and household overcrowding) and/or psychosocial (i.e., single parenthood, psychiatric disorders, and criminality) disadvantages in the parental generation?
3. Do these associations differ by gender across the parental and grandchild generations?

## Methods

### Study population

**Fig. 1:**
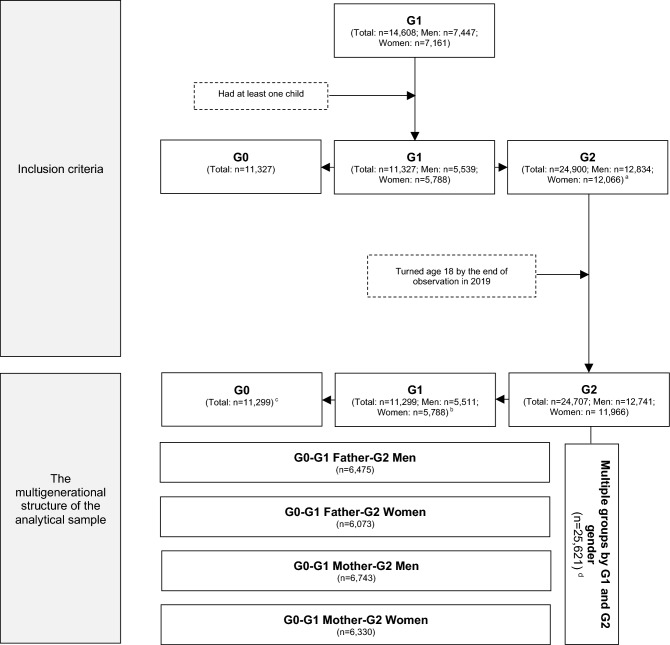
Multigenerational structure of the analytical sample. ^a^ Each individual in the G1 population could contribute with more than one child to the G2 population. ^b^ Refers to either the mother or father of each G2 individual. Note that for a small proportion of G2 individuals, both parents were part of the original SMS cohort members born in 1953. ^c^ Refers to the head of the household in the grandparental generation (i.e., grandparental head of the household on either paternal or maternal side of each G2 individual). ^d^ The discrepancy between this number and the number presented in the previous step is caused by the small proportion of G1 individuals sharing a biological child. In such cases, the G2 individual is counted twice (as the lineages are still different).

The current study is based on the Stockholm Birth Cohort Multigenerational Study (SBC Multigen), which was created through two anonymized longitudinal data materials: RELINK53 and the Stockholm Metropolitan Study (SMS)^21^. SBC Multigen is defined as all individuals born in 1953 and were living in the greater Stockholm metropolitan area in 1963 from the SMS, who were probability matched to the follow-up nationwide register data from RELINK53 (G1, parental generation, n = 14,608), as well as multigenerational linkages. The Regional Ethical Review Board in Stockholm approved the creation of RELINK53 and the SBC Multigen (no. 2017/34-31/5; 2017/684-32). Our study has been performed in accordance with the Declaration of Helsinki, and informed consent is not applicable due to the anonymized nature of the data material used. We used a cohort study design with data for three generations, and the analytical sample for the current study was selected based on inclusion criteria that G1s had at least one child and children of G1s had turned age 18 by the end of observation in 2019. Each G1 individual born in 1953 is treated as a “lineage root” linked to their own parents and children, and their partners are not included in the design. This yielded a sample consisted of 25,621 family lines by G1 and G2 gender, including 11,299 individuals born in 1953, their 22,598 parents (G0, grandparental generation; birth years: 1877–1941), and 24,707 children (G2, grandchild generation; birth years: 1968–2001). Ethical approval of the current study was granted by the Swedish Ethical Review Authority (no. 2021–00,090). Details on the multigenerational structure of the analytical sample are shown in Fig. 1.

### Measures

#### Psychiatric disorders among adult G2s

Psychiatric disorders among G2s, the outcome of interest (n = 1821), were defined as inpatient care due to a main or contributing diagnosis of mental and behavioral disorders from age 18 to 30 (from 1986 until 2019) [Chapter F in the International Classification of Diseases, 10th revision (ICD 10), and the corresponding chapters in the 9th revision (ICD 9)], excluding disorders with an early onset and a strong biological heritage (ICD 10: F70-F98 and corresponding ICD 9 codes, such as attention deficit hyperactivity disorders (ADHD) and intellectual disabilities) that did not co-occur with other diagnoses of mental and behavioral disorders in the National Patient Register (part of RELINK53). These excluded disorders have been found to have higher heritability estimates than most other psychiatric disorders, suggesting that a larger proportion of the observed variability in these disorders is linked to genetic differences^22^. This approach aligns with this study’s primary aim of examining the environmentally-mediated pathways (e.g., parental socioeconomic and psychosocial factors) linking grandparental socioeconomic disadvantages to grandchild psychiatric disorders. Among G2s with psychiatric diagnoses characterized by an early onset and a strong biological heritage, 59.7% (111 out of 186) of them also had records of psychiatric inpatient care with other diagnoses. These G2 individuals were therefore still included in our main analyses.

#### Socioeconomic disadvantages among adult G0s

For G0s, we created the binary variable family-level low income by categorizing the highest total net income from either G1s’ father or mother into two groups characterized by at or below (including individuals who had no income) versus above the lowest 25th percentile in 1963 from the Register of population and income (part of the SMS). Non-employment was defined as head of the household being economically inactive or temporarily not at work (i.e., housewives, studies, military service, illness and disablement, child birth, others) in 1960, and household overcrowding was defined as household having more than two persons per room (kitchen not included) in 1960, both of which were measured at the family level as binary variables and obtained from the 1960 Census (part of the SMS).

#### Socioeconomic and psychosocial disadvantages among adult G1s

For G1s, we used all the above-mentioned dimensions of socioeconomic disadvantages as for G0s, although measured at the individual level with data from different sources, i.e., low income constructed with disposable income in 1990 from the Longitudinal Integration Database for Health Insurance and Labour Market Studies (LISA, part of RELINK53), non-employment and overcrowding extracted from the 1990 Census (part of RELINK53). Regarding individual-level psychosocial disadvantages among G1s, we included single parenthood (i.e., single father or mother) in 1990 from the LISA. Psychiatric disorders (same operationalization as for G2s) and criminality (i.e., prosecution decisions from a court judgment or outside the court) were also included and measured when G2s aged at or below age 18 from the National Patient Register and National Crime Register (part of RELINK53). Descriptive statistics and missing data are presented in Table 1.

**Table 1:** Descriptive statistics and missing data for socioeconomic and psychosocial disadvantages among the grandparental (G0) and parental (G1) generations, stratified by parental (G1) and grandchild (G2) gender.

Socioeconomic and psychosocial disadvantages (Variable type)	G1 Father								G1 Mother							
	G2 Men				G2 Women				G2 Men				G2 Women			
	G0		G1		G0		G1		G0		G1		G0		G1	
	Cases	Missing	Cases	Missing	Cases	Missing	Cases	Missing	Cases	Missing	Cases	Missing	Cases	Missing	Cases	Missing
	n (%)	n (%)	n (%)	n (%)	n (%)	n (%)	n (%)	n (%)	n (%)	n (%)	n (%)	n (%)	n (%)	n (%)	n (%)	n (%)
Low income (binary variable)	1672 (25.82%)	NA	1483 (22.90%)	74 (1.14%)	1507 (24.81%)	NA	1282 (21.11%)	96 (1.58%)	1607 (23.83%)	NA	1661 (24.63%)	96 (1.42%)	1614 (25.50%)	NA	1530 (24.17%)	124 (1.96%)
Non-employment (binary variable)	510 (7.88%)	392 (6.05%)	257 (3.97%)	247 (3.81%)	450 (7.41%)	401 (6.60%)	198 (3.26%)	268 (4.41%)	531 (7.87%)	436 (6.47%)	371 (5.50%)	359 (5.32%)	491 (7.76%)	410 (6.48%)	346 (5.47%)	356 (5.62%)
Overcrowding (binary variable)	1072 (16.56%)	439 (6.78%)	313 (4.83%)	663 (10.24%)	993 (16.35%)	432 (7.11%)	272 (4.48%)	599 (9.86%)	1173 (17.40%)	486 (7.21%)	278 (4.12%)	525 (7.79%)	1058 (16.71%)	447 (7.06%)	259 (4.09%)	550 (8.69%)
Single parenthood (binary variable)	NA	NA	221 (3.41%)	74 (1.14%)	NA	NA	177 (2.91%)	96 (1.58%)	NA	NA	1178 (17.47%)	96 (1.42%)	NA	NA	1094 (17.28%)	124 (1.96%)
Psychiatric disorders (binary variable)	NA	NA	546 (8.43%)	NA	NA	NA	477 (7.85%)	NA	NA	NA	453 (6.72%)	NA	NA	NA	422 (6.67%)	NA
Criminality (binary variable)	NA	NA	1358 (20.97%)	NA	NA	NA	1316 (21.67%)	NA	NA	NA	448 (6.64%)	NA	NA	NA	434 (6.86%)	NA

### Statistical analysis

We employed structural equation modeling (SEM) in the context of mediation analysis for three key reasons^23^: (1) SEM simultaneously estimates both measurement and structural models, allowing us to represent grandparental and parental socioeconomic and psychosocial disadvantages as latent constructs indicated by multiple observed variables; (2) full information maximum likelihood (FIML) estimation handles missing data efficiently^24^; and (3) multiple group comparison enables formal tests of mediation effects across groups by G1 and G2 gender. Although modern causal mediation approaches offer alternative estimators, they currently lack straightforward extensions for latent variables. MLF (i.e., maximum likelihood parameter estimates with standard errors approximated by first-order derivatives and a conventional chi-square test statistic) was chosen as the estimator for the final models, given that the data are binary that violate the multivariate normality assumption, and standard errors using MLR (i.e., maximum likelihood parameter estimates with standard errors and a chi-square test statistic that are robust to non-normality and non-independence of observations) cannot be computed^25^.

SEM requires a priori specification of both measurement and structural models. Within the proposed measurement model, the observed family-level low income, non-employment, and overcrowding were expected to be valid indicators of the latent construct “socioeconomic disadvantages” for G0s (SES G0). Likewise, individual-level low income, non-employment, and overcrowding were specified as indicators of the latent construct “socioeconomic disadvantages” for G1s (SES G1). Moreover, individual-level single parenthood, psychiatric disorders, and criminality were considered as indicators of the latent construct “psychosocial disadvantages” for G1s (PSY G1). We first used WLSMV estimator (i.e., weighted least square parameter estimates using a diagonal weight matrix with standard errors and mean- and variance-adjusted chi-square test statistic that use a full weight matrix) to fit a separate measurement model to be able to report meaningful fit indices (see Supplementary Table S1 online), and then used the MLF estimator to fit both the measurement model and structural model simultaneously.

In terms of the structural model, we tested for mediation on whether the association between grandparental socioeconomic disadvantages (SES G0) and grandchild psychiatric disorders (PD G2) was mediated through parental socioeconomic (SES G1) and/or psychosocial factors (PSY G1). We first fitted a general model for the whole analytical sample, and then we performed multiple group analysis across the four groups by G1 and G2 gender (a. G0–G1 father-G2 men: paternal grandparents-father-grandson, b. G0–G1 father-G2 women: paternal grandparents-father-granddaughter, c. G0–G1 mother-G2 men: maternal grandparents-mother-grandson, and d. G0–G1 mother-G2 women: maternal grandparents-mother-granddaughter). We only considered gender differences in the parental and grandchild generation, given that for the grandparental generation, most of the grandmothers were housewives during 1930–1970, and therefore, socioeconomic conditions at the family level (i.e., head of the household’s socioeconomic conditions) might reflect a more complete picture of their existent material resources^26^.

Data were managed using Stata SE 17, all analyses were performed in Mplus Version 8.

## Results

Table 1 summarizes the information on socioeconomic and psychosocial disadvantages for grandparental (G0) and parental (G1) generations, stratified by parental (G1) and grandchild (G2) gender. Regarding socioeconomic disadvantages, as expected, approximately 25% of G0s and G1s had low income. Meanwhile, approximately 7–8% of G0s, and 3–6% of G1s were non-employed, and around 16–18% of G0s and 4–5% of G1s lived in overcrowded households. In terms of psychosocial disadvantages, single parenthood was more prevalent among G1 mothers (around 17%) compared to G1 fathers (around 3%), whereas criminality was more common among G1 fathers (around 21%) compared to G1 mothers (around 6%). Approximately 6–9% of G1s had experience of inpatient care due to psychiatric disorders. Among G2s, substance-related disorders (2–4%), followed by mood disorders (1–2%), were the most common diagnoses in all groups, except for the G0-G1 mother-G2 women group for whom mood disorders (2.24%) were more common than substance-related disorders (1.93%) (see Supplementary Table S2 and S3 online).

**Fig. 2:**
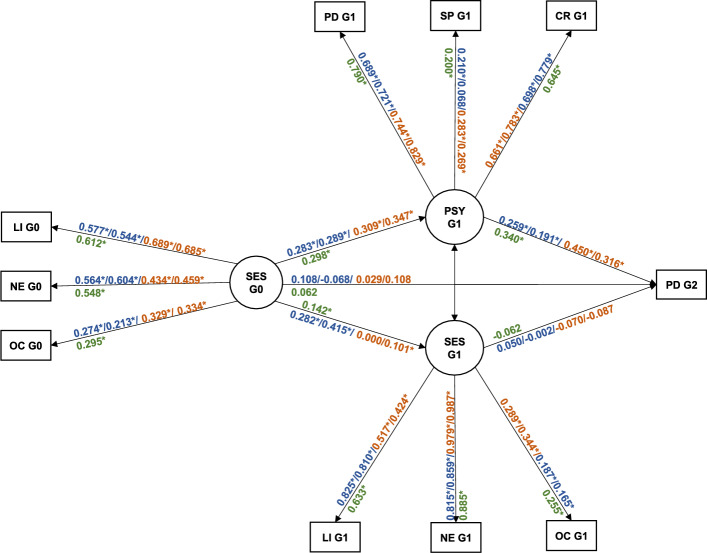
Mediation effects of parental socioeconomic and psychosocial factors in the association between grandparental socioeconomic disadvantages and grandchild psychiatric disorders. Presented estimates are standardized coefficients for the G0-G1 Father-G2 Men/G0-G1 Father-G2 Women/G0-G1 Mother-G2 Men (In blue)/G0-G1 Mother-G2 Women (In orange) from the multiple group model and for the whole analytical sample (In green) from the general model. LI, Low Income; NE, Non-Employment; OC, Overcrowding; SP, Single Parenthood; PD, Psychiatric Disorders; CR, Criminality; SES G0, grandparental socioeconomic disadvantages; SES G1, parental socioeconomic disadvantages; PSY G1, parental psychosocial disadvantages; PD G2, grandchild psychiatric disorders. Parental socioeconomic (SES G1) and psychosocial disadvantages (PSY G1) are significantly correlated with each other (*P* < 0.05, estimates not shown here). * *P* < 0.05.

The final SEM paths tested for the whole analytical sample and multiple groups by G1 and G2 gender with standardized coefficients estimates are presented in Fig. 2. For the whole analytical sample, G0 socioeconomic disadvantages predicted both socioeconomic (β = 0.142) and psychosocial disadvantages (β = 0.298) among G1s. G1 psychosocial disadvantages (β = 0.340), but not G1 socioeconomic disadvantages, predicted G2 psychiatric disorders. In the multiple group analysis, we identified generally similar patterns across the four groups by G1 and G2 gender.

Table 2 presents effect estimates from the mediation models (Table 2a for the whole analytical sample, 2b for the multiple group analysis by G1 and G2 gender). For the whole analytical sample, the standardized total effect of G0 socioeconomic disadvantages on G2 psychiatric disorders was 0.155 (95% confidence interval [CI] 0.099–0.211), and the standardized mediating effect through G1 psychosocial disadvantages was 0.101 (95% CI 0.073–0.130). We did not observe any direct effect from G0 socioeconomic disadvantages to G2 psychiatric disorders, nor any indirect effect through G1 socioeconomic disadvantages. From the multiple group analysis by G1 and G2 gender, we observed total effects of G0 socioeconomic disadvantages on G2 psychiatric disorders across the different gender-specific groups, except for the G0-G1 father-G2 women group, with relatively higher standardized total effect estimates found for the G1-G2 same-sex groups (G0-G1 father-G2 men group: 0.196, 95% CI 0.084–0.307, G0-G1 mother-G2 women group: 0.208, 95% CI 0.101–0.316). We also found mediating effects through G1 psychosocial disadvantages across the different gender-specific groups, except for the G0-G1 father-G2 women group, with the standardized mediating effect magnitude through G1 mothers (G0-G1 mother-G2 men: 0.139, 95% CI 0.078–0.200; G0-G1 mother-G2 women: 0.109, 95% CI 0.047–0.172) being more pronounced.

**Table 2:** Mediation effects of parental (G1) socioeconomic and psychosocial factors in the association between grandparental (G0) socioeconomic disadvantages and grandchild (G2) psychiatric disorders.

Effect	Path	General																			
		Estimate		95% CI		SE		Estimate/SE		P											
**a**. Whole analytical sample																					
Direct effect	SES G0 → PD G2	0.062		− 0.002–0.126		0.033		1.912		0.056											
Indirect effects	SES G0 → SES G1 → PD G2	− 0.009		− 0.020–0.002		0.006		-1.547		0.122											
	SES G0 → PSY G1 → PD G2	0.101		0.073–0.130		0.015		6.894		0.000											
Total effect	0.155	0.099–0.211		0.029		5.424		0.000													

Effect	Path	G0–G1 Father–G2 Men					G0–G1 Fathe–G2 Women					G0–G1 Mother–G2 Men					G0–G1 Mother–G2 Women				
		Estimate	95% CI	SE	Estimate/SE	P	Estimate	95% CI	SE	Estimate/SE	P	Estimate	95% CI	SE	Estimate/SE	P	Estimate	95% CI	SE	Estimate/SE	P
**b.** Multiple groups by parental (G1) and grandchild (G2) gender																					
Direct effect	SES G0 → PD G2	0.108	− 0.019– 0.236	0.065	1.663	0.096	− 0.068	− 0.233– 0.097	0.084	− 0.811	0.417	0.029	− 0.086– 0.145	0.059	0.495	0.621	0.108	− 0.020– 0.235	0.065	1.657	0.098
Indirect effects	SES G0 → SES G1 → PD G2	0.014	− 0.026– 0.054	0.020	0.693	0.488	− 0.001	− 0.074– 0.073	0.038	− 0.018	0.986	0.000	− 0.006– 0.006	0.003	− 0.005	0.996	− 0.009	− 0.026– 0.009	0.009	− 0.993	0.321
	SES G0 → PSY G1 → PD G2	0.073	0.026- 0.120	0.024	3.052	0.002	0.055	− 0.002- 0.112	0.029	1.888	0.059	0.139	0.078– 0.200	0.031	4.485	0.000	0.109	0.047– 0.172	0.032	3.419	0.001
Total effect		0.196	0.084– 0.307	0.057	3.429	0.001	− 0.014	− 0.150- 0.123	0.070	− 0.198	0.843	0.168	0.071– 0.266	0.050	3.384	0.001	0.208	0.101– 0.316	0.055	3.804	0.000

Socioeconomic disadvantages included low income, non-employment, and household overcrowding, and psychosocial disadvantages included single parenthood, psychiatric disorders, and criminality. All estimates are standardized.

*SES G0* grandparental socioeconomic disadvantages, *SES G1* parental socioeconomic disadvantages, *PSY G1* parental psychosocial disadvantages, *PD G2* grandchild psychiatric disorders, *CI* confidence interval, *SE* standard error.

## Discussion

We used multigenerational data to investigate the association between grandparental socioeconomic disadvantages and grandchild psychiatric disorders, and whether this association is mediated through socioeconomic and/or psychosocial factors in the parental generation, and differs by gender across the parental and grandchild generations. Our study has yielded three main results.

First, the major finding of our study is that grandparental socioeconomic disadvantages were associated with grandchild psychiatric disorders, and this association was mediated through psychosocial disadvantages (i.e., single parenthood, psychiatric disorders, and criminality) in the parental generation. Possible explanation could be that grandparental socioeconomic disadvantages may strengthen psychological stressors and susceptibility to crime among their children (i.e., the parental generation) through family adversity (e.g., harsh parenting and child abuse, reduced levels of parental care, and low attachment to parents) and school problems (e.g., lack of provision of educational materials, educational underachievement, and suspension from school)^11,27^. Meanwhile, grandparental socioeconomic disadvantages may also affect their children’s family structure when they grow up through intergenerational transmission of family structure, given that poverty and single parenthood often co-occur, with the odds of poverty being seven times higher for single-parent families than two-parent families^9^. Consequently, the parental generation may, in turn, reproduce the vulnerability process through intergenerational transmission of psychosocial risks while bringing up their own children^28^, which may further have implications on their children’s mental health. Regarding gender differences, relatively high total effect estimates were observed for the parent–child same-sex groups (i.e., paternal grandparents-father-grandson, maternal grandparents-mother-granddaughter). Plausibly, fathers and mothers in the intermediate generation may be more likely to have closer ties with, and receive more socioeconomic influences from their own parents^29^, and they may also sex-type their investments in their children of the same gender^30^, thereby potentially affecting children’s mental health. Moreover, we found the magnitude of mediating effects through mothers’ psychosocial disadvantages in the intermediate generation (i.e., maternal grandparents-mother-grandson, maternal grandparents-mother-granddaughter) to be more pronounced. This implied that maternal psychosocial factors played a significant role in transmitting grandparental socioeconomic influences onto grandchild mental health, possibly due to genetic factors, mothers’ closer emotional bonds and more direct caregiving roles with their offspring compared to fathers^31,32^.

Interestingly, we did not find any mediation effect through parental socioeconomic disadvantages. This suggests that parental socioeconomic disadvantages may lie on the causal pathway of grandparental socioeconomic disadvantages to parental psychosocial disadvantages, or on the pathway of parental psychosocial disadvantages to grandchild psychiatric disorders. This hypothesis could be tested with a serial mediation model in future studies, which would require some temporal distinction between the parental socioeconomic and psychosocial factors. Consistent with this finding, prior research^33^ has reported lower mental health risks among individuals exposed only to material deprivation compared with those facing combined material and psychosocial adversities, suggesting that when including both socioeconomic and psychosocial aspects of disadvantages in the model, socioeconomic disadvantages alone may play a less relevant role in later mental health. This finding could also be interpreted taking into consideration of the Swedish context, in that the expanding welfare state contributed to a historical trend of declining relevance of parental socioeconomic conditions in offspring mental health. Education expansion and changing labor market had led to relative gains of income among the socially disadvantaged groups during the 1870s and the 1970s^34^. Particularly in the 1970s, welfare policies on part-time work, publicly financed child care, and parental leave provided buffers against stress and economic hardships associated with disadvantaged groups^35^, thereby reducing the influences of parental socioeconomic conditions on offspring mental health.

Lastly, we did not observe evidence of direct effect of grandparental socioeconomic disadvantages on grandchild psychiatric disorders. Current available studies on whether or not there exists such a direct effect have reported mixed results. Some studies have found independent effects of maternal grandparental education on grandchild eating disorders, after adjusting for parental education, income, and social^3,36^, and effects of grandfathers’ income^37^ and occupational status^38^ on grandchild cognitive ability and internalizing/externalizing behaviors, independent of parental socioeconomic circumstances. By contrast, other research reveals no evidence of direct effect of grandparental education on grandchildren’s cognitive ability^39^, and no evidence of independent effect of grandparental income on grandchild eating disorders after adjusting for parental socioeconomic positions^36^. Such inconsistencies could be possibly due to issues such as cultural variations, diverse family structures, and methodological biases, which warrant future research for more accurate insights.

### Methodological considerations

The main strength of this study is that we prospectively collected data from local and national surveys and registers that span across three generations, which enabled the measurement of socioeconomic and psychosocial disadvantages, as well as psychiatric disorders in a largely comparable way. Meanwhile, we implemented latent constructs of socioeconomic and psychosocial disadvantages that could represent their multidimensional nature and reduce the measurement error.

We also acknowledge that the findings should be viewed in light of some limitations. First, it has to be noted that our study is constrained by having to make the best use of the available data. For example, because education variable in the 1960 census yielded around 70% of grandparents to be labelled in the low education group (i.e., neither head of the household nor wife has any upper secondary education), we had to exclude education as one of the indicators for the latent construct socioeconomic disadvantages among both the grandparental and parental generations in order to maintain a consistent measurement framework for comparability. Future work in more recent cohorts may consider to include education as an indicator for constructing latent variable socioeconomic status. Second, regarding our outcome psychiatric disorders, we only used data from the inpatient register to ensure adequate data coverage for the grandchild population, which means our analysis was restricted to the most severe cases that required hospitalization. The number of hospital discharges in the inpatient register has remained stable since 1973, while the outpatient register only commenced in 2001 and the prescribed drug register was established in 2005. This means that, for the oldest grandchild—who turned age 33 in 2001 and 37 in 2005—information from ages 18 to 33–37 would be missing^40^. Another drawback is the possibility of socioeconomic differences in how individuals seek mental health treatments. Third, while many psychiatric disorders are considered to have a hereditary component^22^, we have no access to genetic information, and thus it is impossible to rule out biological predisposition of grandchild psychiatric disorders. Specifically, previous research has reported that the association between educational attainment—often linked to socioeconomic background—and mental health appears to have a genetic aetiology^41^. Therefore, we cannot disentangle whether the observed association between grandparental socioeconomic disadvantages and grandchild psychiatric hospitalization is driven in part by inherited genetic liability. Although we excluded disorders with an early onset and a strong biological heritage that did not co-occur with other diagnoses of mental and behavioral disorders (e.g., ADHD when not co-occurring with other diagnoses) from our analyses to reduce genetic confounding, we acknowledge that socioeconomic factors, such as parental unemployment, relative income poverty, and low educational attainment, are still relevant in increasing children’s risk of ADHD^42^. Fourth, although our study offers initial insight into how grandparental socioeconomic disadvantages may relate to grandchild psychiatric disorders via parental psychosocial disadvantages, they do not permit formal causal inference. Future research could consider applying counterfactual-based causal mediation techniques to more rigorously decompose direct and indirect effects under clearly stated causal assumptions^43^. Lastly, comparisons with previous studies could be compromised by the lack of consistency in the measurement between the latent variables in our study and the observable variables used elsewhere, although the consistency in the magnitude and direction of the associations remains remarkable.

## Conclusion

In conclusion, we found an association between grandparental socioeconomic disadvantages and grandchild psychiatric disorders, which was mediated through parental psychosocial disadvantages but not through parental socioeconomic disadvantages. The mediation was more pronounced via psychosocial disadvantages among mothers as opposed to fathers. These findings suggest that addressing parental psychosocial disadvantages, particularly among mothers, is important for mitigating social inequalities in mental health as they evolve across generations.

## Supplementary Information

Supplementary Information.

## Data Availability

The data underlying this article cannot be shared publicly due to ethical regulations for the Stockholm Birth Cohort Multigenerational Study (SBC Multigen). The data will be shared on reasonable request to the corresponding author, who will forward it to the steering committee of the SBC Multigen, and the committee will evaluate the request and decide on data to extract for specific purposes.
