# Supplementary material for: Grandparental socioeconomic disadvantages and grandchild psychiatric disorders: the mediating role of parental socioeconomic and psychosocial factors
Source: Sci Rep. 2025 Jun 20;15:20120. doi: 10.1038/s41598-025-04282-z (PMC12181403; doi:10.1038/s41598-025-04282-z)
Supplement: Supplementary file 1 — Supplementary Information. [file 41598_2025_4282_MOESM1_ESM.docx]

**Table S1 Standardized factor loadings and fit statistics for the measurement model**

|  | Standardized factor loadings | P-Value |
| --- | --- | --- |
| **SES G0** |  |  |
| LI G0 | 0.891 | 0.000 |
| NE G0 | 0.380 | 0.000 |
| OC G0 | 0.234 | 0.000 |
| **SES G1** |  |  |
| LI G1 | 0.634 | 0.000 |
| NE G1 | 0.954 | 0.000 |
| OC G1 | 0.203 | 0.000 |
| **PSY G1** |  |  |
| SP G1 | 0.045 | 0.012 |
| PD G1 | 0.783 | 0.000 |
| CR G1 | 0.650 | 0.000 |

Note: WLSMV estimator was applied in estimating the measurement model.

LI, Low Income; NE, Non-Employment; OC, Overcrowding; SP, Single Parenthood; PD, Psychiatric Disorders; CR, Criminality; SES G0, grandparental socioeconomic disadvantages; SES G1, parental socioeconomic disadvantages; PSY G1, parental psychosocial disadvantages; PD G2, grandchild psychiatric disorders.

Fit indices:

RMSEA (Root Mean Square Error of Approximation): 0.038

CFI: 0.855

TLI: 0.800

SRMR (Standardized Root Mean Square Residual): 0.068

**Table S2 Descriptive statistics (occurrence and proportions)** **for the outcome grandchild psychiatric disorders by categories, stratified by parental (G1) and grandchild (G2) gender**

| Grandchild psychiatric disorders by categories | G1 Father | | G1 Mother | |
| --- | --- | --- | --- | --- |
|  | **G2 Men** | **G2 Women** | **G2 Men** | **G2 Women** |
| **Organic disorders** | 3 (0.05%) | 3 (0.05%) | 6 (0.09%) | 3 (0.05%) |
| **Substance-related disorders** | 256 (3.95%) | 158 (2.60%) | 254 (3.77%) | 122 (1.93%) |
| **Schizophrenic/ Psychotic disorders** | 38 (0.59%) | 29 (0.48%) | 35 (0.52%) | 26 (0.41%) |
| **Mood disorders** | 86 (1.33%) | 123 (2.03%) | 88 (1.31%) | 142 (2.24%) |
| **Anxiety disorders** | 49 (0.76%) | 93 (1.53%) | 44 (0.65%) | 78 (1.23%) |
| **Personality disorders** | 14 (0.22%) | 42 (0.69%) | 23 (0.34%) | 46 (0.73%) |
| **Mental retardation and diseases originating in childhood ^a^** | 36 (0.56%) | 27 (0.44%) | 27 (0.40%) | 22 (0.35%) |
| **Other disorders** | 39 (0.60%) | 64 (1.05%) | 37 (0.55%) | 77 (1.22%) |

^a^ Cases were only included if there were other co-occurring diagnoses of psychiatric disorders

**Table S3 Psychiatric disorders: ICD-9 and ICD-10 codes**

| **Disorders** | **Conditions** | **ICD-9 code** | **ICD-10 code** |
| --- | --- | --- | --- |
| Organic disorders | Senile and pre-senile psychotic conditions | 290.0–290.9 | F00–F09 |
|  | Transient organic psychotic conditions | 293.0, 293.1, 293.8, 293.9 |  |
|  | Other organic psychotic conditions | 294.0, 294.1, 294.8, 294.9 |  |
| Substance-related disorders | Alcoholic psychoses | 291.0–291.9 | F10–F19, F55 |
|  | Drug psychoses | 292.0–292.9 |  |
|  | Alcohol dependence | 303.0–303.9 |  |
|  | Drug dependence | 304.0–304.9 |  |
|  | Nondependent abuse of drugs | 305.0–305.9 |  |
| Schizophrenic/ Psychotic disorders | Schizophrenia | 295.0–295.9 | F20–F29 |
|  | Psychotic | 298.8–298.9 |  |
|  | Paranoia | 297.1–297.3 |  |
| Mood disorders | Bipolar | 296.0–296.1, 296.4–296.8 | F30, F31, F34.0 |
|  | Depression | 296.2, 296.3, 300.4, 311 | F32, F33, F34.1, F38.1 |
|  | Other | 296.9 | F34.8, F34.9, F38.0, F38.8, F39 |
| Anxiety disorders | Anxiety | 300.0, 300.2, 300.3, 309.8 | F40, F41, F42 |
|  | Acute stress | 308.3 | F43.0, 43.1, 43.8, 43.9 |
| Personality disorders | Personality disorders | 301.0–301.9 | F60, F61, F62, F69 |
| Other disorders | | 309.0–309.4, 309.8–309.9,  302.0–302.9, 297.0–297.3, 297.8–297.9, 300.1, 300.5, 300.6, 300.7, 300.8, 300.9, 308.0–308.2, 308.9, 310 | F43.2, F99  F52, F64, F65, F66  F44, F48, F50, F51, F53, F54, F68 |

Source: Canadian Institute for Health Information, Hospital Mental Health Services in Canada, 2005-2006 Ottawa 2007, p. 27-28 (<https://www150.statcan.gc.ca/n1/pub/82-622-x/2011006/tbl/tbla-eng.htm>).
